# Ethnic disparities in out–of–pocket expense on medicines in Peru: Evidence from a nationwide survey

**DOI:** 10.1016/j.puhip.2023.100442

**Published:** 2023-10-28

**Authors:** Jerry K. Benites-Meza, Liseth Pinedo-Castillo, Miguel Cabanillas-Lazo, Percy Herrera-Añazco, Benoit Mougenot, Vicente A. Benites-Zapata

**Affiliations:** a Sociedad Científica de Estudiantes de Medicina de la Universidad Nacional de Trujillo, Trujillo, Peru; b Grupo Peruano de Investigación Epidemiológica, Unidad de Investigación para la Generación y Síntesis de Evidencias en Salud, Universidad San Ignacio de Loyola, Lima, Peru; c Asociación Científica de Estudiantes de Medicina de la Universidad Señor de Sipán, Chiclayo, Peru; d Sociedad Científica de San Fernando, Lima, Peru; e Facultad de Medicina de San Fernando, Universidad Nacional Mayor de San Marcos, Lima, Peru; f Universidad Privada del Norte, Trujillo, Peru; g Facultad de Ciencias Empresariales, Universidad San Ignacio de Loyola, Lima, Peru; h Centro de Excelencia en Investigaciones Económicas y Sociales en Salud, Universidad San Ignacio de Loyola, Lima, Peru; i Unidad de Investigación para la Generación y Síntesis de Evidencias en Salud, Vicerrectorado de Investigación, Universidad San Ignacio de Loyola, Lima, Peru; j Red Internacional en Salud Colectiva y Salud Intercultural, Mexico City, Mexico

**Keywords:** Health expenditures, Medical economics, Ethnic groups, Pharmacies, Health surveys, Peru (source: NLM/MeSH)

## Abstract

**Background:**

Despite improvements in health insurance coverage, out-of-pocket (OOP) health spending remains a public health issue in Peru, and OOP payment has implications for disease treatment in ethnic minorities. We aimed to analyze the ethnic disparities in the OOP payment and estimate the gaps related to observable risk factors in the OOP payment on medicines by ethnic conditions during 2014–2016 in Peru.

**Study design:**

cross-sectional study.

**Methods:**

We conducted a secondary data analysis using the National Health User Satisfaction Survey. The outcome was the participants' OOP payment in self-reported medications. Ethnic minorities were considered participants who habitually spoke a language other than Spanish at home. Crude and adjusted linear regression models were performed, and the Oaxaca-Blinder decomposition method was used to assess the OPP payment differential by ethnic minority condition, explained by their individual and sociodemographic characteristics.

**Results:**

We analyzed 11,346 surveyed, the mean age was 40.78 years, and 57.67 % were women. There was lower OOP payment in medications among ethnic minorities in the adjusted analysis (Beta coefficient [β]: −0.11; 95 % confidence interval [95%CI]: −0.21 to −0.01; p = 0.043). In the Oaxaca-Blinder decomposition analysis, a gap of 0.19 USD in the OOP payment in medicines among ethnic minorities was found (p < 0.001), and the explained component by the variables measured in this research only represents 40.5 % of the gap (p = 0.001).

**Conclusion:**

There was less OOP expenditure on medicines in ethnic minorities. However, the measured variables explain only 40.5 % of these gaps. Therefore, we recommend future research that measures other variables that explain aspects of OOP spending on medicines not identified in this research. Likewise, our findings can be used to establish policies with an intercultural approach that adapt health documents to native languages or are disseminated by trained people from their communities.

**What this study adds**.

- Out-of-pocket (OOP) health spending remains a public health issue in Peru, has implications for disease treatment in ethnic minorities
- In a secondary data analysis using a national representative Survey, we found that there was less OOP expenditure on medicines in ethnic minorities, and the measured variables explain almost half of these gaps
- Policies are needed to improve access of ethnic minority patients to health care facilities

**Implications for Policy and Practice**.

- The utility of OOP is probably limited in population with lower use of health services.
- Lower utilization of health services is probably due to difficulties in access, discrimination, mistreatment, and language difficulties may be among them
- It is necessary to propose intercultural strategies to increase the use of health services.

## 1 Introduction

The out-of-pocket (OOP) health spending is defined as the direct payment made by individuals to health care providers at the time of service use [1]. The OOP expense may include payment for medical fees, payment for procedures, purchase of medications and supplies, use of home remedies, co-payments, and deductibles paid by those with insurance [1]. As well as in various Latin American countries, where OOP spending can constitute a public health problem [2], despite the increase in health insurance coverage during the last years [3], the OOP cost is still a public health issue in Peru. A national study showed that OOP payment increased between 2010 and 2014 for both members and non-members of a health system [4].

Various reasons explain the increase in OOP spending in our country. For example, poor households incorporated older adults into the insurance system, which increased the declaration of chronic diseases within the home and with it the need for more expenses for their care [1]. Despite improvements in insurance coverage, poor households preferred not to go to health facilities and opted to search for apothecaries and pharmacies and their OOP expenses grew more proportionally compared to the increase in their total expenses [1]. That is, this group was experiencing less financial protection despite being insured [1]. Likewise, the reduction in poverty in households with insurance allowed them to incur greater expenses and made the decision to incur OOP expenses easier when a health problem occurred [1].

As in the general population, OOP expenses also affects vulnerable people such as the elderly. Another study showed that, in 2017, 56.5 % of older adults had OOP payments [5], which suggests that other vulnerable populations in the country could still have OOP payments despite improved health insurance coverage. In Latin America, people from ethnic minorities are considered vulnerable groups, subject to higher social exclusion and discrimination [[6], [7], [8], [9]]. This situation affects some health indicators, reduces the well-being of the individual in the initial stage of development and decreases their socioeconomic achievements in the middle and long term [[6], [7], [8], [9]]. For example, in 2015, a national survey showed that, compared to the non-indigenous population, the Quechua and Aymara speaking population suffered from more chronic diseases (33.7 %, 43.8 % and 58.6 % respectively) [10]. However, they were more likely to have the health insurance that the government provides to those with fewer resources, the Universal Health Insurance (SIS), compared to the non-indigenous population, where health insurance for the population with more resources predominates, the social security (Essalud) [10].

The ethnic diversity observed in the region has several and complex implications for identifying the ethnic condition of the individual. One of the primary indicator used for identifying ethnicity is based on spoken language from indigenous people. Language is the main manifestation of people's attachment to their norms and culture, a specific set of questions about languages have been included in national surveys for many years [[11], [12]].

Several studies revealed that OOP payment has implications for disease treatment in ethnic minorities. A study showed that OOP payment influenced adherence to adjuvant endocrine therapy for breast cancer in the United States, and this adherence varied by ethnicity [13]. Another study showed that compared to White non-Hispanic, ethnic minorities had significantly lower OOP expenses for diabetes management per year when adjusted for sociodemographic characteristics and comorbidities [14].

In Peru, even though health insurance coverage has improved [3], it is still deficient, as expressed in OOP spending as a problem both in the general population, in vulnerable populations such as the elderly and aspects such as spending on the purchase of drugs at the national level [1,[4], [5], [15]]. Although there are studies that have evaluated aspects related to OOP expense in ethnic minorities [[13], [14]], to our knowledge, they have not evaluated whether OOP spending is significant, nor the proportion of observable risk factors that affect OOP payment in ethnic minorities in a country, where 55 indigenous peoples and 48 native languages have been registered [[16], [17]]. Therefore, the objective of the present study was to analyze the ethnic disparities in the OOP payment and estimate the gaps related to observable risk factors in the OOP payment in drugs by ethnic conditions at the national level during the 2014–2016 period.

## 2 Methods

### 2.1 Study design

Secondary analysis of the fourth questionnaire of the National Health User Satisfaction Survey 2014–2016 (ENSUSALUD) of the National Institute of Statistics and Informatics of Peru (INEI) and The Peruvian National Health Superintendency (SUSALUD) [18]. ENSUSALUD was a nationally representative survey whose objective was to collect information about the functioning and performance of the Health Services Providers (IPRESS), which allows the identification and measurement of the perception and satisfaction of the main authors (external and internal users) of external consultation, health insurance, apothecaries and pharmacies, emergency personnel and management. The data collection method was through direct interviews with personnel duly trained for this purpose by the INEI [18].

### 2.2 Population, sample and sampling

The study population was composed of people aged 15 years or older who came to buy some medicine for themselves, their partner or child in a pharmacy or apothecary close to the IPRESS at the national level. Sampling was performed in 181 Health Services Providers, thereby conducting a probabilistic, stratified, and two-staged selection [18].

The primary sampling unit was the IPRESS of the Ministry of Health and regional governments (MINSA-GR), the Social Health Insurance in Peru (EsSalud), the Health of the Armed Forces and the Police (Health Services), and the private sector clinics (PSC) which were randomly selected. The secondary unit was the users of apothecaries and pharmacies selected in a non-probabilistic way for convenience. Twenty-five strata were corresponding to the 25 political regions of Peru. Based on this, the expansion factors were estimated. It is important to note that the inference is limited to the care in the outpatient medical consultation of the IPRESS in the country [18].

### 2.3 Eligibility criteria

**Fig. 1:**
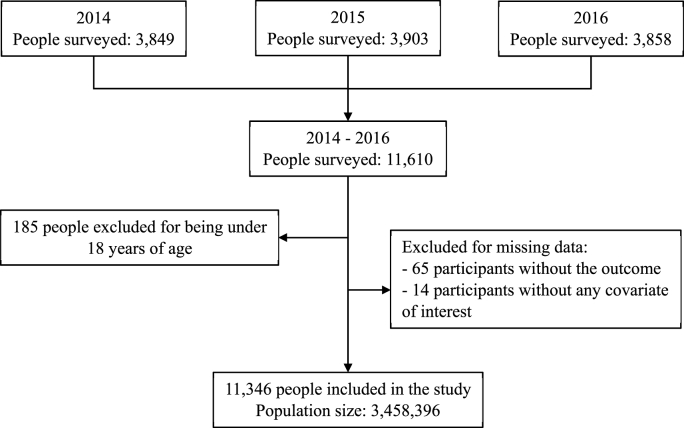
Flowchart of the selection of participants included in the analysis, ENSUSALUD 2014–2016.

The fourth questionnaire survey of the ENSUSALUD from 2014 to 2016 included a total of 11,610 surveyed users. Users aged 18 years and older in a pharmacy or apothecary close to a health care facility were included in the analysis. The participants with missing data in the outcome variable (0.6 %) or any of the covariates of interest (0.1 %) were excluded. The final calculated sample size was 11,346 participants, representing an expanded population of 3,458,396 people (Fig. 1).

### 2.4 Variables

The outcome of interest was the OOP payment in self-reported medications by participants. The interviewing personnel had access to the purchased medicines, corroborating the information on OOP spending on medicines. Since the information on out-of-pocket spending on medicines was provided in the national currency of Peru (Soles), we converted it to the USD using the average exchange rate during the years 2014–2016, which was 3.13. For the respective analysis, the natural logarithm of this variable was considered.

The main independent variable was language. This was evaluated through the question: What is the language in which you communicate at home? The response options were: Spanish, Quechua, other. For the analysis, this variable was dichotomized into Spanish and Quechua/others.

**Fig. 2:**
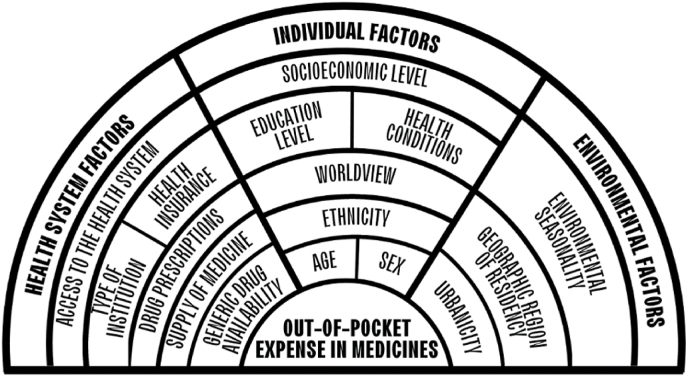
Framework of variables related to out-of-pocket payments for medicines.

The confounding variables were: sex, age (18–39, 40 to 59, 60 or more), higher education (yes, no), employment status (unemployment, dependent, independent), health insurance (yes, no), available medical prescription (yes, no), type of institution (apothecary, pharmacy), level of care (I level, II level, III level) and region of residence (metropolitan of Lima, others). The authors developed a framework of measured and unmeasured variables in this study that relate to OOP expense on drugs (Fig. 2).

The level of care refers to the category of health sector institutions according to their levels of complexity. Together, they determine their resolution capacity, responding to similar socio-health realities and designed to face equivalent demands [19].

### 2.5 Ethical aspects

Our investigation did not require approval by an institutional review board or ethics review committee because ENSUSALUD is a free access database belonging to SUSALUD and available at http://portal.susalud.gob.pe/blog/category/base-de-datos/. In ENSUSALUD no identification was recorded on the identity of each participant, guaranteeing the confidentiality of the information provided by the participants. In addition, all potential participants gave their verbal consent to be surveyed. In Peru there are not regulations for the development of observational studies, only for clinical trials [20]. Therefore, the ethical flow of these studies is based by international regulations. In this sense, secondary data analyzes correspond to the category of studies exempt from review by an ethics committee.

### 2.6 Statistical analysis

The ENSUSALUD databases corresponding to the years 2014, 2015 and 2016 were downloaded in SPSS format, then exported and analyzed with STATA v15.0 (TX, StataCorp LP). All analyzes considered the complex sample design of ENSUSALUD, using the *svy* command.

In the descriptive analysis, the categorical variables were expressed in frequencies and percentages, their respective 95 % CI. The numerical variables are presented as averages with 95 % CI. To determine whether there are significant differences between the independent variables according to the language, the Chi-square test with Rao Scott correction for complex sampling was used. To determine the differences between numerical variables, we performed the Wald test.

Linear regression models were constructed to calculate β coefficients with their respective 95 % CI to determine the association between the logarithm of OOP expenses in medications and language. The first model was a crude regression, while the second model was adjusted for confounding variables according to an epidemiological approach. To assess collinearity, we determined variance inflation factors (VIF). Additionally, stratified models were presented according to sex, age (less than or older than 60 years old) and level of care. A statistically significant p-value less than 0.05 was considered.

Finally, to estimate the gaps related to observable risk factors in the OOP payment in drugs due to ethnic conditions at the national level during the 2014–2016 period, the Oaxaca-Blinder (OB) decomposition method was used. The OB decomposition procedure divides the OOP expense differential into two groups, one part explained by the differential characteristics of each group that, we assume, have some impact on the risk factor, such as educational level or place of residence; and a residual part that cannot be explained by these differences in the endowments of individuals. The unexplained part includes group differences on unobservable predictors (residual) and is usually used as a measure of discrimination [21].

## 3 Results

The average age was 40.78 years, 57.67 % of the participants were women, 29.25 % lived in Metropolitan Lima, 47.34 % had higher education, 40.95 % was unemployed and 66.52 % had health insurance. In addition, 85.24 % bought their medications at pharmacies and 29.99 % bought them with a prescription. In addition, 49.89 % belonged to second-level care health centers. Only 2.11 % reported speaking Quechua or another language. The average out-of-pocket expense for medicines was 5.62 (5.40–5.84) USD (Table 1).

**Table 1:** General characteristics of apothecaries and pharmacies users, ENSUSALUD 2014–2016 (n = 11,346; N = 3,458,396).

Characteristics	Absolute frequency	Weighted proportion^a^	
	n	%	95 % CI
**Out-of-pocket expense in medicines (USD)**			
Mean (95 % CI)	5.62	5.40–5.84	
**Language**			
Spanish	11053	97.89	97.60–98.14
Quechua/Others	293	2.11	1.86–2.40
**Sex**			
Women	6495	57.67	56.79–58.54
Men	4851	42.33	41.45–43.20
**Age (years)**			
Mean (95 % CI)	40.78	40.50–41.07	
18 to 39	6207	53.72	52.75–54.67
40 to 59	3749	33.35	32.43–34.29
60 or more	1390	12.93	12.30–13.58
**Higher education**			
No	6040	52.66	51.27–54.03
Yes	5306	47.34	45.97–48.72
**Employment status**			
Unemployment	4624	40.95	39.86–42.05
Dependent	2090	18.42	17.04–19.89
Independent	4632	40.63	39.51–41.74
**Health insurance**			
No	3549	33.48	32.44–34.53
Yes	7797	66.52	65.47–67.56
**Drug Prescriptions**			
No	8191	79.01	68.28–71.69
Yes	3155	29.99	28.31–31.72
**Type of institution**			
Apothecary	1669	14.76	13.54–16.07
Pharmacy	9677	85.24	83.93–86.46
**Level of care**			
I Level	2232	22.45	19.40–25.85
II Level	7005	49.89	46.40–53.39
III Level	2109	27.65	25.05–30.41
**Geographic region of residency**			
Metropolitan Lima	1509	29.25	26.32–32.37
Other regions	9837	70.75	67.63–73.68

**95%CI:** 95 % Confidence intervals.

a Weights and the design effect of the complex survey sampling were included.

When analyzing the distribution of the drugs, it was found that the most frequently purchased drugs were analgesics/antipyretics/corticosteroids (32.56 %), followed by non-steroidal anti-inflammatory drugs (NSAIDs) (26.35 %) and antibiotics (25.40 %). When analyzing the distribution of the types of drugs according to language, statistically significant differences were found for drugs such as antihistamines/respiratory pathologies (p < 0.001), nutritional supplements (p = 0,023) and drugs for metabolic disorders (p < 0.001) **(**Table 2**)**.

**Table 2:** Types of medicine purchased by users of apothecaries and pharmacies according to language.

Type of medicine purchased by participants	All n = 11,346		Quechua/Other n = 293		Spanish n = 1053		p-value^a^
	n	%	n	%	n	%	
**Antibiotics**							0.110
No	8335	74.60	206	71.02	8129	74.68	
Yes	3011	25.40	87	28.98	2924	25.32	
**NSAIDs**							0.308
No	8258	73.65	208	71.49	8050	73.70	
Yes	3088	26.35	75	28.51	3003	26.30	
**Gastrointestinal**							0.838
No	10055	88.46	266	88.72	9252	88.46	
Yes	1291	11.54	27	11.28	1801	11.54	
**Analgesics/Antipyretics/Corticoids**							0.857
No	7469	67.44	200	67.72	7269	67.44	
Yes	3877	32.56	93	32.28	3789	32.56	
**Antihistamines/Respiratory pathologies**							**<0.001**
No	9518	84.46	266	67.72	9252	67.44	
Yes	1828	15.54	27	32.28	1801	32.56	
**Nutritional supplement**							**0.023**
No	10730	94.37	280	96.61	10450	94.32	
Yes	616	5.63	13	3.39	603	5.68	
**Cardiac pathologies**							0.687
No	10852	95.51	278	95.14	10574	95.51	
Yes	494	4.49	15	4.86	479	4.49	
**Antiparasitic/Antiviral/Antimycotic**							0.061
No	10782	95.18	272	93.89	10510	95.21	
Yes	564	4.82	21	6.11	543	4.79	
**Metabolic disorders**							**<0.001**
No	11116	97.91	290	99.53	10826	97.87	
Yes	230	2.09	3	0.47	227	2.13	
**Neurological pathologies**							0.966
No	10889	95.82	282	95.77	10607	95.82	
Yes	457	4.18	11	4.23	446	4.18	
**Other**							0.494
No	10116	88.54	254	87.58	9862	88.56	
Yes	1230	11.46	39	12.42	1191	11.44	

**95%CI:** 95 % Confidence intervals.

Weights and the design effect of the complex survey sampling were included.

a Refers to the statistical significance obtained from the comparison of the proportions between the categories of the variables considering the complex sampling of the survey.

Bivariate analysis by language showed statistically significant differences for age, higher education, employment status, health insurance, level of care, and geographic region of residence (p < 0.001). We also found statistically significant differences with respect to the average out-of-pocket spending on medicines according to language (p = 0,006) **(**Table 3**)**.

**Table 3:** General characteristics of apothecaries and pharmacies users according to language.

Characteristics	Quechua/Others n = 293			Spanish n = 11,053			p-value^a^
	n	%	95 % CI	n	%	95 % CI	
**Out-of-pocket expense in medicines (USD)**							0,006
Mean (95 % CI)	4.99	4.27–5.72		5.63	5.41–5.86		
**Sex**							0.618
Women	169	58.54	55.15–61.84	6326	57.65	56.76–58.54	
Men	124	41.46	38.16–44.85	4727	42.35	41.46–43.24	
**Age (years)**							**<0.001**
18 to 39	111	39.70	35.46–44.10	6096	54.02	53.05–54.98	
40 to 59	114	35.55	31.60–39.71	3635	33.31	32.37–34.26	
60 or more	68	24.75	21.22–28.65	1322	12.67	12.04–13.33	
**Higher education**							**<0.001**
No	272	91.49	88.37–93.83	5768	51.82	50.44–53.20	
Yes	21	8.51	6.17–11.63	5285	48.18	46.80–49.56	
**Employment status**							**<0.001**
Unemployment	107	37.97	33.70–42.44	4517	41.02	39.92–42.12	
Dependent	16	5.32	3.89–7.23	2074	18.71	17.33–20.17	
Independent	170	56.71	52.43–60.90	4462	40.28	39.16–41.41	
**Health insurance**							**<0.001**
No	57	23.39	19.56–27.72	3492	33.69	32.65–34.76	
Yes	236	76.61	72.28–80.44	7561	66.31	65.24–67.35	
**Drug Prescriptions**							0.149
No	221	73.96	68.21–78.99	7970	69.92	68.18–71.61	
Yes	72	26.04	21.01–31.79	3083	30.08	28.39–31.82	
**Type of institution**							0.470
Apothecary	43	12.76	8.24–19.25	1626	14.80	13.40–16.10	
Pharmacy	250	87.24	80.75–91.76	9427	85.20	83.90–86.40	
**Level of care**							**<0.001**
I Level	59	22.41	18.46–26.92	2173	22.46	19.36–25.90	
II Level	220	69.79	63.86–75.13	6785	49.46	45.94–53.00	
III Level	14	7.80	4.00–14.67	2095	28.08	25.48–30.84	
**Geographic region of residency**							**<0.001**
Metropolitan Lima	4	3.67	1.41–9.22	1505	29.80	26.79–33.00	
Other regions	289	96.33	90.78–98.59	9548	70.20	66.99–73.21	

**95%CI:** 95 % Confidence intervals.

Weights and the design effect of the complex survey sampling were included.

a Refers to the statistical significance obtained from the comparison of the proportions between the categories of the variables considering the complex sampling of the survey.

There was lower OOP payment in medications in participants speaking Quechua or other languages in the crude analysis than those who speak Spanish. In the adjusted analysis, the association was maintained (β: −0.11; 95 % CI: −0.22 to −0.01; p = 0.045). In the analysis stratified by sex, the association was only presented for women (β: −0.12; 95 % CI: −0.23 to −0.02; p = 0.024). In the age-stratified analysis, a significant association was found for those older than 60 years (β: −0.34; 95 % CI: −0.58 to −0.09; p = 0.007). Finally, in the analysis stratified by level of care, the association was only maintained for the first level (β: −0.34; 95 % CI: −0.45 to −0.22; p < 0.001) **(**Table 4**)**.

**Table 4:** Linear regression model for the association between language and spending on medicines and stratified by sex, age, and level of care in adult users of apothecaries and pharmacies.

Exposure		Out-of-pocket expense in medicines					
		Crude Model^a^			Adjusted Model^a^^,^^b^		
		β	95 % CI	p-value	β	95 % CI	p-value
**All sample**	Spanish	Ref.	–	–	Ref.	–	–
	Quechua/Others	−0.19	−0.32 to −0.06	0.006	−0.11	−0.22 to −0.01	**0.045**
**Stratified for Sex**							
Women	Spanish	Ref.	–	–	Ref.	–	–
	Quechua/Others	−0.20	−0.34 to −0.06	0.006	−0.12	−0.23 to −0.02	**0.024**
Men	Spanish	Ref.	–	–	Ref.	–	–
	Quechua/Others	−0.18	−0.34 to −0.01	0.034	−0.08	−0.24 to 0.07	0.293
**Stratified for Age**							
<60 years	Spanish	Ref.	–	–	Ref.	–	–
	Quechua/Others	−0.14	−0.25 to −0.02	0.022	−0.04	−0.13 to 0.05	0.376
≥60 years	Spanish	Ref.	–	–	Ref.	–	–
	Quechua/Others	−0.45	−0.71 to −0.19	0.001	−0.34	−0.58 to −0.09	**0.007**
**Stratified for Level of care**							
I Level	Spanish	Ref.	–	–	Ref.	–	–
	Quechua/Others	−0.55	−0.65 to −0.45	<0.001	−0.34	−0.45 to −0.22	**<0.001**
II Level	Spanish	Ref.	–	–	Ref.	–	–
	Quechua/Others	−0.05	−0.19 to −0.09	0.501	−0.03	−0.16 to 0.09	0.581
III Level	Spanish	Ref.	–	–	Ref.	–	–
	Quechua/Others	−0.43	−0.10 to −0.97	0.108	0.09	−0.23 to 0.41	0.586

**β:** Beta coefficient; **95 % CI:** 95 % Confidence intervals.

a A linear regression model considering the design effect and the weights of the complex sampling of the survey.

b Adjusted for sex, age, higher education, employment status, health insurance, prescription, type of institution, and geographic region of residency.

According to the OB decomposition analysis, a gap of 0.19 USD in the OOP payment in medicines was found, which disadvantages those speaking Quechua or another native language (p < 0.001). The explained component was 0.08, which represents 40.54 % of the gap (p < 0.05). On the other hand, the unexplained component was 0.11, representing the remaining 59.46 % (p < 0.05). In the OB analysis stratified by sex, the explained component of the OOP expenses gap represented 37.71 % for women and 52.78 % for men. Likewise, for older adults, the explained component was only 71.10 %, while adults were 25.18 %. Finally, according to the level of care, the explained component was 38.79 % for the first level, 27.18 % for the second level, and 80.12 % for the third level (Table 5).

**Table 5:** Oaxaca-Blinder decomposition.

Measure		Coefficient	95 % CI	p-value
**All sample**	Average of logarithm of medicines expenditures (Spanish)	1.01	0.98 to 1.05	<0.001
	Average of logarithm of medicines expenditures (Quechua/Others)	0.83	0.70 to 0.95	<0.001
	Average difference	0.19	0.06 to 0.31	0.003
	Explained component	0.08	0.02 to 0.14	0.014
	Unexplained component	0.11	0.01 to 0.22	0.045
	Explained proportion	40.54 %		
	Unexplained proportion	59.46 %		
**Stratified by sex**				
Women	Average of logarithm of medicines expenditures (Spanish)	1.05	1.02 to 1.09	<0.001
	Average of logarithm of medicines expenditures (Quechua/Others)	0.86	0.73 to 0.98	<0.001
	Average difference	0.20	0.07 to 0.32	0.002
	Explained component	0.07	0.01 to 0.14	0.038
	Unexplained component	0.12	0.02 to 0.22	0.019
	Explained proportion	37.71 %		
	Unexplained proportion	62.29 %		
Men	Average of logarithm of medicines expenditures (Spanish)	0.96	0.91 to 1.01	<0.001
	Average of logarithm of medicines expenditures (Quechua/Others)	0.78	0.61 to 0.96	<0.001
	Average difference	0.18	0.01 to 0.35	0.044
	Explained component	0.09	0.02 to 0.17	0.013
	Unexplained component	0.08	−0.07 to 0.24	0.288
	Explained proportion	52.78 %		
	Unexplained proportion	47.22 %		
**Stratified by age**				
<60 years	Average of logarithm of medicines expenditures (Spanish)	0.98	0.95 to 1.02	<0.001
	Average of logarithm of medicines expenditures (Quechua/Others)	0.85	0.73 to 0.96	<0.001
	Average difference	0.14	0.02 to 0.25	0.018
	Explained component	0.10	0.03 to 0.16	0.003
	Unexplained component	0.04	−0.05 to 0.13	0.371
	Explained proportion	71.10 %		
	Unexplained proportion	28.90 %		
≥60 years	Average of logarithm of medicines expenditures (Spanish)	1.21	1.13 to 1.29	<0.001
	Average of logarithm of medicines expenditures (Quechua/Others)	0.76	0.51 to 1.01	<0.001
	Average difference	0.45	0.20 to 0.70	0.001
	Explained component	0.11	0.03 to 0.20	0.011
	Unexplained component	0.34	0.11 to 0.57	0.005
	Explained proportion	25.18 %		
	Unexplained proportion	74.82 %		
**Stratified by level of care**				
I Level	Average of logarithm of medicines expenditures (Spanish)	0.96	0.90 to 1.02	<0.001
	Average of logarithm of medicines expenditures (Quechua/Others)	0.41	0.30 to 0.52	<0.001
	Average difference	0.55	0.42 to 0.68	<0.001
	Explained component	0.21	0.15 to 0.28	<0.001
	Unexplained component	0.34	0.21 to 0.46	<0.001
	Explained proportion	38.79 %		
	Unexplained proportion	61.21 %		
II Level	Average of logarithm of medicines expenditures (Spanish)	0.91	0.87 to 0.95	<0.001
	Average of logarithm of medicines expenditures (Quechua/Others)	0.86	0.73 to 1.00	<0.001
	Average difference	0.05	−0.09 to 0.18	0.477
	Explained component	0.01	−0.03 to 0.06	0.565
	Unexplained component	0.04	−0.09 to 0.16	0.581
	Explained proportion	27.18 %		
	Unexplained proportion	72.82 %		
III Level	Average of logarithm of medicines expenditures (Spanish)	1.23	1.18 to 1.29	<0.001
	Average of logarithm of medicines expenditures (Quechua/Others)	1.67	1.19 to 2.14	<0.001
	Average difference	−0.43	−0.90 to 0.31	0.066
	Explained component	−0.35	−0.65 to −0.04	0.028
	Unexplained component	−0.09	−0.36 to 0.19	0.527
	Explained proportion	80.12 %		
	Unexplained proportion	19.88 %		

## 4 Discussion

The main results of the present study show lower OOP expenses in medicines among the participants speaking Quechua or other languages, the gap being greater in women, among persons over 60 years old, and among those who belong to the first level of care. However, the reasons for these findings were explained only 40.5 % by the observable differences of the variables considered.

In recent years, coverage of access to health insurance has grown significantly in Peru, facilitating access to more excellent protection for people of diverse socioeconomic and ethnic conditions [3]. Health insurance is relevant in a country where regions with ethnic minorities still have disadvantages related to health. For example, women from ethnic minorities such as Quechua and Aymara present disadvantages in different indicators of maternal and child health [[22], [23]]. Similarly, mistreatment when using health services has been documented in Quechua, Asháninca, and Nomatsiguengas patients and patients from the Mantaro Valley in the central Andes Mountains of Peru [[24], [25]]. Health insurance in Peru increased from 60.5 % in 2008 to 76.4 % in 2017. In this way, SIS coverage, insurance for the population with fewer resources, increased from 34 % in 2008 to 47 % in 2017 [3]. Being a woman increased the possibility of affiliation to the SIS, while being between 18 and 39 years old, residing in Metropolitan Lima, and being non-poor reduced this possibility [3]. This increase in coverage was not homogeneous; in the departments where many ethnic minorities live, this coverage was less than 50 % in 2017 [3].

This evidence perhaps explains that OOP payment in health in Peru is still an issue in purchasing drugs. One study estimated that, on average, OOP expenses in drugs were US$ 8.14 in 2007, compared to US$ 9.68 in 2016 [15]. This apparent discrepancy between more excellent insurance coverage in Peru and an increase in OOP payments may be because a greater demand for services due to the extension of insurance to the SIS highlighted the weakness of the service offer and probably that this was deteriorating [1].

However, according to our results, there are fewer OOP expenses for ethnic minorities. Nevertheless, it has been reported that there is ineffective access to medicines in the vast majority of health establishments, despite having accessed outpatient care [26]. Therefore, access to the physician is achieved but not to the necessary medicines leading to an increase in OOP payment. This apparent discrepancy may be associated with some hypotheses that we will raise later.

Unlike our study, other authors have found higher OOP costs in some ethnic minorities. For example, a study in the United States showed that Mexicans had a higher proportion of OOP payments than Whites after adjusting for socioeconomic and demographic factors [27]. Another study in the same country showed that being African-American lowered costs for Medicare-managed diabetes care, although it increased the disparity between total healthcare costs and OOP expenses [28]. In contrast, another study showed that racial and ethnic disparities were initially observed between non-Hispanic Whites and Hispanics and between non-Hispanic Whites and non-Hispanic Blacks in the OOP expenses ratio of older adults in the United States. However, these disparities disappeared after controlling by utilization or health needs [29].

In contrast, these studies were carried out in a specific health system and considered different ethnic minorities from those evaluated in our research; however, some aspects were found in these studies and may explain our findings. Probably, as observed in the study of older adults, the use of health services is also a variable to consider [29]; in that sense, this apparent decrease in OOP expenses in the Peruvian ethnic minorities could be due to a lower use of health services. Indeed, it has been reported that, in the rural population, which is the area where a large part of these minorities live, more than 60 % do not seek medical attention [30].

Likewise, another study that evaluated the non-use of formal health services found that 62 % in the Highlands and 56 % in the Amazonian region do not use health services despite needing them [31], territories where ethnic minorities frequently live in Peru. Although various reasons explain it, including aspects such as distance and difficulties in acceding health centers in rural areas, discrimination, among other reasons, language may also be a factor to consider [32]. A study observed that Latinos in the United States were less likely to use drugs compared to Whites; it was found that one of the most critical factors for this disparity was limited English proficiency [27]. These differences in language proficiency could also explain why people over 60 years of age had fewer OOP expenses, as they were probably less prone to proficiency in Spanish and therefore made less use of health services. On the contrary, it is also probable that the greater use of health services and the greater probability of obtaining care decreases the OOP payment for women [33].

However, our findings are explained to warrant a more in-depth analysis of unobservable characteristics. Our results showed that less than 40 % of the gap was explained by our variables for women, while for older adults, it was about 25 %. The unexplained component of the gap in the OOP expenses for drug purchase was higher than the component explained by observable characteristics. These results contrast with a study that evaluated the use of different medications by comparing groups by the different ethnic conditions, including Hispanics, non-Hispanic Afro-descendants, and non-Hispanic whites [34]. This study showed that observable characteristics mainly explained the differences in the use of therapeutic drugs for Afro-descendants and Whites. Although our study used a nationally representative survey, our analysis was subject to pre-established variables, limiting the possibility of analyzing other unobservable factors that explain our findings. Although there are studies that have evaluated the issue in other ethnic minorities [13,14], to our knowledge, no studies have evaluated the ethnic differences in OOP expense in medicines for ethnic minorities present in Peru [16]. This point is relevant because, for many ethnic minorities, the concept of health is linked to the community beyond the individual and includes an essential spiritual element firmly related to the well-being of the ecosystem and the planet [35]. These elements, associated with their worldview, have not been evaluated and perhaps are part of the unobservable characteristics that explain our findings.

According to the level of care, the component explained with the variables studied was about 27 % in the second level and 39 % in the first level. According to the technical standard of the categories of health establishments of the MINSA, the first level of care includes health centers attended by non-medical or medical professionals, including hospitalization services, for the care of less complex problems. In the case of second-level establishments, general or specialized care hospitals are included [19,36]. Although we cannot establish which aspects the unexplained component includes according to the level of care, they may include variables related to some of the characteristics of the health care of Quechua and Aymara patients. Although, according to the 2019 National Household Survey, 82.2 % and 68.3 % of the Quechua and Aymara populations, respectively [37], had health insurance, there are still problems in the use of health services, which would increase the probability of not using them and increase the OOP. A study showed that, although patients belonging to Amazonian peoples used MINSA's unpaid dental services, Aymara patients paid for them, probably in private services [38]. Although various structural and cultural barriers prevent the use of health services, such as distance from establishments or lack of health personnel of the same ethnic group [39], an additional factor is mistreatment [32]. Another study showed that being a Quechua patient or speaking a native language doubled, and being of Aymara ethnicity tripled the probability of not going to health services due to abuse [32]. Although these studies evaluated all health services, regardless of the level of care, it is likely that these barriers are similar at all levels of health and perhaps more so in second-level services, where problems such as obstetric-gynecology are treated. Indeed, for example, delivery care in the Aymara population of Puno includes beliefs [40] that make them more likely not to use the free health services of MINSA and therefore increase OOP.

Although OOP spending can be used to indicate some aspects of health systems, our findings suggest that, although observable aspects only explain less than half of the causes, its usefulness is probably limited in a population with lower use of health services. Furthermore, although the causes are diverse, lower utilization of health services due to difficulties in access, discrimination, mistreatment, and language difficulties may be among them [[29], [30], [31], [32],^39^]. In this sense, it is necessary to propose strategies to increase the use of health services.

It is important to mention that the regression analyses did not include participants' income level as a confounding variable. Income level may be related to ethnicity and is also an essential factor in utilizing medical services and the ability to pay for medications. Not considering this factor will likely result in underestimating the magnitude of ethnic disparities in OOP expenses on medicines and could further explain these gaps.

Despite the fact that increasing the number of health facilities in remote areas may be a good strategy, intercultural policies should be implemented. In this regard, the Pan American Health Organization suggests some recommendations for developing a licensing and accreditation system for intercultural health services that could also be used in other Latin American countries with these problems [41]. These recommendations include recognizing a holistic vision of health while respecting spirituality, that the vulnerability of indigenous peoples is acknowledged and that health systems recognize the existence of traditional medicine as equally valid, and that they develop mechanisms of interrelation between them [41]. Other recommendations include providing mechanisms for the empowerment of indigenous peoples and creating spaces for participation between governments and indigenous communities to facilitate decision-making about health and that quality standards be made based on their needs [41]. It is also advised to implement aspects of traditional medicine in intercultural bilingual education and to establish intercultural facilitators in health facilities [41]. Although, to the best of our knowledge, no studies have shown the effectiveness of intervention strategies to improve indigenous peoples' access to health services in Latin America [42], experiences in the United States and Canada show that they can be successful [43].

The present study has certain limitations. First, as secondary data analysis, it lacks a specific design to address the research question. Second, the analysis is based on a single health indicator through OOP payments for medicine. Third, the analysis measures the person's ethnic status based on language, with the possibility that we have underestimated the proportion of people from these ethnic minorities. Fourth, missing data in some variables could represent a potential selection bias. However, the proportion of missing values is very small and would not significantly affect the results of our study [44]. Fifth, the income level variable was not included, which, as discussed, could have affected the results. Therefore, we suggest including income level as a confounding variable in future studies. Sixth, our analysis is limited by the use of data from 2014 to 2016; therefore, there is a possibility of a variation in out-of-pocket healthcare spending over time up to 2023. Unfortunately, the survey has not been conducted again, making it impossible to analyze a period beyond 2016. However, an analysis measuring trends in out-of-pocket healthcare spending from 2017 to 2020 in a nationally representative sample does not show differences, even for the COVID-19 period [45]. Therefore, despite the age of our data, a similar conclusion would be obtained if replicated our analysis using current data. Finally, many of the variables were self-reported, which could lead to the misclassification of the data.

In conclusion, there was less out-of-pocket expenditure on medicines in those ethnic minorities, the gap being greater in women and those over 60 years old. However, the observable differences explain only 40.5 % of these gaps.

We recommend following the intercultural approach that adapts documents to native languages or is disseminated by trained people from their communities to identify unobservable elements that explain the health aspects of ethnic minorities in our country [46].

## Statements of ethical approval

This study analyzed a secondary database that collected data without identifiers and did not violate the integrity of the participants. Access to the database was free and available on the ENSUSALUD website.

## Funding

This research did not receive any specific grant from funding agencies in the public, commercial, or not-for-profit sectors.

## Authors' contributions

Conceptualization: Percy Herrera-Añazco, Benoit Mougenot and Vicente A. Benites-Zapata; Data curation: Jerry K. Benites-Meza, Liseth Pinedo-Castillo and Miguel Cabanillas-Lazo; Formal analysis: Jerry K. Benites-Meza, Benoit Mougenot and Vicente A. Benites-Zapata; Methodology: Jerry K. Benites-Meza, Benoit Mougenot and Vicente A. Benites-Zapata; Writing – original draft: Jerry K. Benites-Meza, Percy Herrera-Añazco and Vicente A. Benites-Zapata; Writing – review & editing: Jerry K. Benites-Meza, Liseth Pinedo-Castillo, Miguel Cabanillas-Lazo, Percy Herrera-Añazco, Benoit Mougenot and Vicente Benites-Zapata.

## Declaration of competing interest

The authors declare that they have no known competing financial interests or personal relationships that could have appeared to influence the work reported in this paper.
